# A Chromosome-Scale Assembly of the Garden Orach (*Atriplex hortensis* L.) Genome Using Oxford Nanopore Sequencing

**DOI:** 10.3389/fpls.2020.00624

**Published:** 2020-05-25

**Authors:** Spencer P. Hunt, David E. Jarvis, Dallas J. Larsen, Sergei L. Mosyakin, Bozena A. Kolano, Eric W. Jackson, Sara L. Martin, Eric N. Jellen, Peter J. Maughan

**Affiliations:** ^1^Department of Plant and Wildlife Sciences, Brigham Young University, Provo, UT, United States; ^2^M.G. Kholodny Institute of Botany, National Academy of Sciences of Ukraine, Kyiv, Ukraine; ^3^Institute of Biology, Biotechnology and Environmental Protection, Faculty of Natural Sciences, University of Silesia in Katowice, Katowice, Poland; ^4^25:2 Solutions, Rockford, MN, United States; ^5^Agriculture and Agri-Food Canada, Ottawa Research and Development Centre, Ottawa, ON, Canada

**Keywords:** *Amaranthaceae*, *Atriplex hortensis*, Hi-C, orach, orphan crop, proximity-guided assembly

## Abstract

*Atriplex hortensis* (2*n* = 2*x* = 18, 1C genome size ∼1.1 gigabases), also known as garden orach and mountain-spinach, is a highly nutritious, broadleaf annual of the *Amaranthaceae-Chenopodiaceae* alliance (*Chenopodiaceae* sensu stricto, subfam. *Chenopodioideae*) that has spread in cultivation from its native primary domestication area in Eurasia to other temperate and subtropical regions worldwide. *Atriplex* L. is a highly complex but, as understood now, a monophyletic group of mainly halophytic and/or xerophytic plants, of which *A. hortensis* has been a vegetable of minor importance in some areas of Eurasia (from Central Asia to the Mediterranean) at least since antiquity. Nonetheless, it is a crop with tremendous nutritional potential due primarily to its exceptional leaf and seed protein quantities (approaching 30%) and quality (high levels of lysine). Although there is some literature describing the taxonomy and production of *A. hortensis*, there is a general lack of genetic and genomic data that would otherwise help elucidate the genetic variation, phylogenetic positioning, and future potential of the species. Here, we report the assembly of the first high-quality, chromosome-scale reference genome for *A. hortensis* cv. “Golden.” Long-read data from Oxford Nanopore’s MinION DNA sequencer was assembled with the program Canu and polished with Illumina short reads. Contigs were scaffolded to chromosome scale using chromatin-proximity maps (Hi-C) yielding a final assembly containing 1,325 scaffolds with a N50 of 98.9 Mb – with 94.7% of the assembly represented in the nine largest, chromosome-scale scaffolds. Sixty-six percent of the genome was classified as highly repetitive DNA, with the most common repetitive elements being Gypsy-(32%) and Copia-like (11%) long-terminal repeats. The annotation was completed using MAKER which identified 37,083 gene models and 2,555 tRNA genes. Completeness of the genome, assessed using the Benchmarking Universal Single Copy Orthologs (BUSCO) metric, identified 97.5% of the conserved orthologs as complete, with only 2.2% being duplicated, reflecting the diploid nature of *A. hortensis*. A resequencing panel of 21 wild, unimproved and cultivated *A. hortensis* accessions revealed three distinct populations with little variation within subpopulations. These resources provide vital information to better understand *A. hortensis* and facilitate future study.

## Introduction

*Atriplex hortensis* L. (2*n* = 9*x* = 18), also known as garden orach or mountain-spinach, is a highly nutritious, leafy annual plant. It is a moderately xero-halophytic species that is resistant to salinity, a wide range of temperatures, and drought. Originating in Eurasia, *A. hortensis* has been a minor vegetable food source in multiple areas of the Trans-Himalayan region and has since become naturalized throughout the Americas. It exhibits incredible variation in pigmentation as a result of its variable content of betalains, as well as substantial differences in height and seed production (Tanaka et al., 2008; Simcox and Stonescu, 2014).

*Atriplex hortensis* has been recognized for its medicinal properties which were shown to improve digestion, increase circulation and boost the immune system (Rinchen et al., 2017). Additionally, *A. hortensis* has been used in land rehabilitation projects because of its ability to establish well, grow rapidly, reduce soil erosion and compete with native plants (McArthur et al., 1983; Simon et al., 1994; Wright et al., 2002). As a result, *A. hortensis* is important for both domestic and wild browsing animals where other forage crops are lacking. Despite its affinity for low to moderate saline areas where it has little competition from non-halophytes, *A. hortensis* can also grow where total soluble salts are low, making it well suited to a multitude of different environments (Welsh and Crompton, 1995).

As the world continues to search for new ways to feed its ever-growing population, new food sources have gained popularity that have helped provide diversity to diets while capitalizing on less desirable, underutilized or even fallow landscapes for agriculture. Given its xero-halophytic characteristics, *A. hortensis* is an intriguing candidate for contributing to world food security, especially in areas rich in saline soils. In comparison to other leafy vegetable crops, *A. hortensis* seeds and leaves are both edible and have protein contents of 26% (dry weight) in seeds, which is comparable to some legumes (Wright et al., 2002), and 35% (dry weight) in leaves, which is higher than spinach (*Spinacea oleracea* L.), a close relative of *A. hortensis* also belonging to the same subfamily *Chenopodioideae*, but to a different tribe (*Anserineae*; see Fuentes-Bazan et al., 2012). However, the seeds contain antinutritional saponins that must be removed by washing and/or seed abrasion. In this respect *A. hortensis* resembles its distant relative quinoa (*Chenopodium quinoa* Willd.); the name recently formally proposed for nomenclatural conservation (Mosyakin and Walter, 2018), which also contains saponins. Interestingly, sweet varieties of quinoa have been identified that have a nonsense mutation in the regulator of the saponin biosynthetic pathways (Jarvis et al., 2017) – suggesting similar pathways could be targeted to remove antinutritional saponins in *A. hortensis*. The seeds of *A. hortensis* have higher fat, ash, fiber and lysine contents than most cereal grains (Wright et al., 2002). Its high protein content, which includes an essential amino acid profile that meets the WHO and UN-FAO recommended adult levels, also makes *A. hortensis* very attractive as a novel protein source.

*Atriplex hortensis* belongs to the family *Chenopodiaceae* in the strict sense, which is now often included in the extended family *Amaranthaceae* sensu lato; this group (*Chenopodiaceae*+*Amaranthaceae*) is phylogenetically nested in the core clade of the order *Caryophyllales*, which in turn, belongs to core eudicots, the largest and most diverse clade of angiosperms (for an overview of high-level phylogeny of the group, see Hernández-Ledesma et al., 2015; APG IV, 2016, and references therein).

The merger of the traditionally recognized families *Chenopodiaceae* and *Amaranthaceae* sensu stricto into one family under the priority name *Amaranthaceae* sensu lato proposed already in the first version of the APG system (APG (Angiosperm Phylogeny Group), 1998) remained unchanged in all other APG modifications (see, APG IV, 2016, and references therein). It was widely followed by many researchers and users of botanical nomenclature, but usually not by the experts in taxonomy of *Chenopodiaceae* (s. str.), who mainly continued to accept the two families. Not discussing here the reasons of and arguments for the two concepts of familial and subfamilial delimitation in the *Amaranthaceae*/*Chenopodiaceae* alliance (which will be discussed in a separate article, now in progress), we, however, note that the merger of the two families resulted in some confusion and miscommunication in recent literature regarding the usage of family names, and especially names of infrafamilial suprageneric entities (such as subfamilies and tribes). For example, some authors use the subfamily name *Amaranthoideae* in its traditional sense for just one group in *Amaranthaceae* s. str., while others may use it to cover all formerly recognized groups in *Amaranthaceae* s. str. (including *Amaranthoideae*, *Gomphrenoideae*, etc.). To avoid any uncertainty, we conventionally use in the present article the following nomenclature (both formal and informal names): (1) the group uniting *Chenopodiaceae* and *Amaranthaceae* s. str. (forming together the extended *Amaranthaceae* sensu APG) is referred to under an informal designation “*Amaranthaceae*/*Chenopodiaceae* aliance;” (2) the family-rank names *Amaranthaceae* and *Chenopodiaceae* refer to the groups corresponding to the two traditionally recognized families; and (3) the sufbamily-rank name *Chenopodioideae* (in paralel with other recognized subfamilies, such as *Betoideae*, *Salsoloideae*, etc.) corresponds to just one group of *Chenopodiaceae* s. str., but not to the group covering the whole family *Chenopodiaceae* in its traditional circumscription; similarly, *Amaranthoideae* refers to the subfamily-rank subdivision of *Amaranthaceae* s. str., comparable to *Gomphrenoideae*.

Recent molecular phylogenetic and taxonomic studies have led to considerable improvements in taxonomy and in our understanding of phylogenetic relationships in the order *Caryophyllales* in general and *Atriplex* and its closer relatives in particular (Kadereit et al., 2010; Zacharias and Baldwin, 2010; Brignone et al., 2019; Morales-Briones et al., 2020). However, few molecular studies have been focused specifically on *A. hortensis* in recent years.

As it is viewed now, *Atriplex* is nested in the larger clade corresponding to the tribe *Atripliceae* (including *Chenopodieae*, which is the correct name for the group if placed in *Chenopodiaceae*, not *Amaranthaceae* s.l.) as outlined by Fuentes-Bazan et al. (2012), and/or to a smaller clade corresponding to the tribe *Atripliceae* in a narrower sense, as outlined by Kadereit et al. (2010). The *Atripliceae* in the narrow sense is sister to another clade (informally called *Chenopodieae* I; see Kadereit et al., 2010) containing *Chenopodium* s. str. (including Australian *Rhagodia* R. Br. and *Einadia* Raf.; see Fuentes-Bazan et al., 2012; Mosyakin and Iamonico, 2017) in its much restricted sense, excluding taxa formerly placed in *Chenopodium* sensu lato but now recognized in phylogenetically more distant genera *Blitum* L. (which is close to *Spinacia* L.), *Chenopodiastrum* S. Fuentes, Uotila & Borsch, *Dysphania* R. Br., *Lipandra* Moq., *Oxybasis* Kar. & Kir., and *Teloxys* Moq. (see Fuentes-Bazan et al., 2012; Hernández-Ledesma et al., 2015).

The clade of *Atripliceae* (sensu Kadereit et al., 2010) contains two main subclades (informally named as the *Archiatriplex*-clade and *Atriplex*-clade) with several smaller lineages, some of which are currently recognized as separate genera. As circumscribed now, the phylogenetically coherent and monophyletic *Atriplex* includes several groups that were earlier described and recognized as separate genera, such as *Obione* Gaertn. and some Australian and North American groups. Despite morphological distinctiveness of some of those groups, they are phylogenetically deeply rooted in *Atrilpex* and thus their recognition as separate genera is not recommended. In contrast, several genera are recognized in the *Archiatriplex*-clade, namely *Archiatriplex* G.L.Chu, *Exomis* Fenzl ex Moq., *Extriplex* E.H. Zacharias, *Grayia* Hook. & Arn., *Holmbergia* Hicken, *Manochlamys* Aellen, *Microgynoecium* Hook. f., *Proatriplex* (W.A.Weber) Stutz & G.L. Chu, and *Stutzia* E.H. Zacharias (Kadereit et al., 2010; Brignone et al., 2019). They represent relicts of earlier diversification events in the group. Also, some additional early-branching (“basal”) lineages of the *Atriplex*-clade can also probably be recognized as separate genera. For example, in addition to the currently recognized genera *Halimione* Aellen and *Atriplex* s. str. (Kadereit et al., 2010), such groups as *Cremnophyton* Brullo & Pavone from Malta containing *C. lafrancoi* Brullo & Pavone (=*Atriplex lafrancoi* (Brullo & Pavone) G. Kadereit & Sukhorukov; see Kadereit et al., 2010) and the mainly Central Asian *Sukhorukovia* Vasjukov (2015) with *S. cana* (C.A. Mey.) Vasjukov (*Atriplex cana* C.A. Mey. = *Cremnophyton canum* (C.A. Mey.) G.L. Chu) may be probably assigned the generic rank after further research.

Since *A. hortensis* is the nomenclatural type of the genus, it naturally belongs to *Atriplex* subgen. *Atriplex* sect. *Atriplex* (Art. 22.1 of the *International Code for Nomenclature of algae, fungi and plants*: Turland et al., 2018). This section houses at least two other species, *A. sagittata* Borkh. (earlier often known under the synonymous name *A. nitens* Schkuhr) and *A. aucheri* Moq., which seem to be most closely related to *A. hortensis* (Sukhorukov, 2006). The clade of *A. hortensis* and its two close relatives belongs to the grade of early-branching clades of *Atriplex* s. str. containing taxa with C_3_ photosynthesis (Brignone et al., 2019).

The geographic and taxonomic origins of domesticated *A. hortensis* remain elusive because at present the species is known mostly (or exclusively?) in cultivated and escaped (and locally naturalized) populations. It probably originated somewhere within the geographic ranges of its closest relatives, in Central Asia or adjacent regions, or it could be native in the Mediterranean region and/or Asia Minor (Sukhorukov, 2014).

Although recent studies have tested the limits of salt-tolerance of *A. hortensis* (Vickerman et al., 2002; Sai Kachout et al., 2011), there has been little to no research conducted to develop genetic tools necessary for accelerating *A. hortensis* breeding. One phenotypic characteristic in need of improvement for seed production is the panicle, which consists of two types of flowers usually mixed on the same plant. One type produces 3–5 mm diameter seed that are encased within large, papery bracteoles that are not retained well under windy conditions at maturity. The other flower type produces 1–2 mm black fruits/seeds that have no bracteoles but are instead subtended by easily removed tepals.

To better understand the underlying genetic basis of the xero-halophytic, nutritive and unique pigmentation characteristics of *A. hortensis*, and to more accurately assess phylogenetic relationships within its family and genus, we sequenced the *A. hortensis* genome. We show that ultra-long reads produced by the portable, real-time Oxford Nanopore Technology (Oxford, United Kingdom) MinION sequencing system (Lu et al., 2016) with short-read polishing and chromatin-contact mapping is an effective approach to generate a high-quality genome assembly in a moderately large and complex genome of a diploid plant species. We annotated the genome with a deeply sequenced transcriptome from various *A. hortensis* plant tissues, and we demonstrate the quality of the chromosome-level genome assembly and annotation using Benchmarking Universal Single-Copy Orthologs (BUSCO) (Simão et al., 2015) to assess the completeness of the assembled genome. Genomic comparison to other Caryophyllales within the *Amaranthaceae-Chenopodiaceae* family identified highly syntenic and orthologous chromosomal relationships. Together, these resources provide an initial, important foundation for accelerated genetic improvement to neodomesticate this potentially valuable crop.

## Materials and Methods

### Plant Material

*Atriplex hortensis* cv. “Golden” was obtained from Wild Garden Seed (Philomath, Oregon) and used for whole-genome sequencing and assembly. Sterilized seed were grown hydroponically in a growth chamber at BYU. An 11-h photoperiod was maintained using broad-spectrum light sources. Growing temperatures ranged from 18°C (night) to 20°C (day). Hydroponic growth solution, changed weekly, was made from MaxiBloom® Hydroponics Plant Food (General Hydroponics, Sevastopol, CA, United States) at a concentration of 1.7 g/L.

The resequencing panel consisted of 21 A. hortensis accessions: 15 from the United States Department of Agriculture collection (USDA, ARS, NALPGRU;^1^); five each from two separate commercial seed vendors (Baker Creek Heirloom Seed Company, Mansfield, Missouri and Wild Garden Seed, Philomath, Oregon); and one accession collected in the wild in Utah (BYU 1317 from Park City, Utah). Plants used in the resequencing panel were originally collected from across Europe (France, Poland, countries of the former Soviet Union, former Serbia/Montenegro and Norway) and North America (United States, and Canada). A complete list of all plant materials including passport information is provided in Table 1.

**TABLE 1:** Identification and passport information for plant materials used for the genome sequencing and the resequencing panel.

Accession	Source	Improvement status (name)	Collection location	Latitude/Longitude	Sequencing technology	SRR number^2^
**Resequencing Panel**						
BYU 1317	BYU herbarium	Wild	Park City, UT, United States	40.66796, −111.515032	Illumina^3^	SRR11123184
Red Orach	Baker Creek Heirloom Seeds	Improved	Mansfield, MO, United States	N/A^3^	Illumina	SRR11123183
PI 310383	USDA	Uncertain	Tashkent, Uzbekistan	N/A	Illumina	SRR11123172
PI 323313	USDA	Improved	Poland	N/A	Illumina	SRR11123170
PI 345962	USDA	Uncertain	Norway	N/A	Illumina	SRR11123169
PI 357340	USDA	Improved (Zolta)	Former Serbia/Montenegro	41.91667000, 22.41667000	Illumina	SRR11123168
PI 357342	USDA	Improved (Zolta Prilepska)	Former Serbia/Montenegro	41.34640000, 21.55440000	Illumina	SRR11123167
PI 357344	USDA	Improved (Lokalna Zolta)	Former Serbia/Montenegro	41.81200000, 21.99470000	Illumina	SRR11123166
PI 357346	USDA	Improved (Gradinarska)	Former Serbia/Montenegro	41.57920000, 21.57190000	Illumina	SRR11123165
PI 357347	USDA	Improved (Debarska)	Former Serbia/Montenegro	41.52500000, 20.52750000	Illumina	SRR11123164
PI 370353	USDA	Improved (Lokalna)	Former Serbia/Montenegro	41.89890000, 21.40810000	Illumina	SRR11123182
PI 370354	USDA	Improved (Mestna)	Former Serbia/Montenegro	41.94140000, 21.41280000	Illumina	SRR11123181
PI 372512	USDA	Wild	Alberta, Canada	51.4502063, −112.7061764	Illumina	SRR11123180
PI 379088	USDA	Improved (2261)	Former Serbia/Montenegro	41.84890000, 21.82030000	Illumina	SRR11123179
PI 379093	USDA	Improved (2475)	Former Serbia/Montenegro	41.38250000, 22.28750000	Illumina	SRR11123178
PI 379095	USDA	Improved, (Skopska)	Former Serbia/Montenegro	42.00000000, 21.43330000	Illumina	SRR11123177
PI 420154	USDA; 218	Wild (218)	France	N/A	Illumina	SRR11123176
Triple Purple	Wild Garden Seed Co.	Improved	Philomath, OR, United States	N/A	Illumina	SRR11123174
P1reselection	Wild Garden Seed Co.	Improved	Philomath, OR, United States	N/A	Illumina	SRR11123173
P6 reselection	Wild Garden Seed Co.	Improved	Philomath, OR, United States	N/A	Illumina	SRR11123171
Golden	Wild Garden Seed Co.	Improved	Philomath, OR, United States	N/A	Illumina	SRR11123175
**Whole Genome Sequencing**						
Golden	Wild Garden Seed Co.	Improved	Philomath, OR, United States	Leaf tissue	Oxford Nanopore	SRR11147376
**Hi-C scaffolding**						
Golden	Wild Garden Seed Co.	Improved	Philomath, OR, United States	Chicago^TM^ Hi-C	Illumina	SRR11147368
Golden	Wild Garden Seed Co.	Improved	Philomath, OR, United States	HiRiSE^TM^ Hi-C	Illumina	SRR11147367
**Transcriptome**						
Golden	Wild Garden Seed Co.	Improved	Philomath, OR, United States	Root tissue	Illumina	SRR11147369
Golden	Wild Garden Seed Co.	Improved	Philomath, OR, United States	Stem tissue	Illumina	SRR11147370
Golden	Wild Garden Seed Co.	Improved	Philomath, OR, United States	Floral tissue	Illumina	SRR11147371
Golden	Wild Garden Seed Co.	Improved	Philomath, OR, United States	Leaf tissue	Illumina	SRR11147372
Golden	Wild Garden Seed Co.	Improved	Philomath, OR, United States	Whole plantlet	Illumina	SRR11147373
Golden	Wild Garden Seed Co.	Improved	Philomath, OR, United States	Root tissue – NaCl treated^4^	Illumina	SRR11147374
Golden	Wild Garden Seed Co.	Improved	Philomath, OR, United States	Leaf tissue – NaCl treated	Illumina	SRR11147375

*Accessions of *A. hortensis* originated throughout Europe and North America. For the whole genome sequencing, Hi-C scaffolding and transcriptome data, the source tissue and/or library type is provided in the Latitude/Longitude column. ^1^Meters above sea level. ^2^Sequence read archive accession number for each resequenced line. Maintained by national Center for Biotechnology Information (https://dataview.ncbi.nlm.nih.gov/objects?linked_to_id=PRJNA607334). ^3^N/A indicates no data. ^4^Treated with 350 mM NaCl (see section “Materials and Methods”).*

### DNA Extraction, Library Preparation, and Oxford Nanopore Sequencing

The Golden variety of *A. hortensis* was grown hydroponically in a growth chamber at BYU as previously described. Plants were dark-treated for 72 h at which point young leaf tissue was harvested and extracted for high molecular weight (HMW) genomic DNA using the Qiagen (Germantown, MD) Genomic-tip protocol. The DNA concentration was checked using the dsDNA High Sensitivity DNA Assay on the Qubit^®^ 2.0 Fluorimeter (Invitrogen, Merelbeke, Belgium).

Samples for DNA sequencing were prepared with and without fragmentation using Covaris g-TUBEs (Woburn, MA) and the ZYMO DNA Clean & Concentrator-5 column (Irvine, CA, United States). Samples were fragmented using both the ZYMO DNA kit and Covaris g-TUBEs following manufacturer’s instructions. Samples prepared with the Covaris g-TUBEs were fragmented at several centrifugation speeds, including 3,800, 4,000, and 4,200 RPM. In total, nine libraries from the original DNA stock were prepared for sequencing using the 1D Genomic DNA by Ligation MinION library preparation kit. Libraries were sequenced on R9 flow cells on a MinION for 48 h using MinKNOW 2.0 software with the following settings: DNA, PCR-free, no multiplexing, SQK-LSK109 kit (Oxford Nanopore Technologies, Ltd., Oxford, United Kingdom). No alterations were made to voltage or time. Albacore v2.3.1, part of the MinKNOW package, was used for base calling.

### Read Cleaning, Draft Genome Assembly, and Polishing

MinIONQC (Lanfear et al., 2019) was used with default settings to summarize sequence data. NanoFilt (De Coster et al., 2018) was then used to trim and filter reads using the following options: −*q* = 8, headcrop = 25, −l = 2000. Porechop v.0.2.3 (Wick et al., 2017) was used to trim adaptors from sequence data with the default options. Draft genomes were assembled using multiple assemblers, specifically Canu v.1.7.1 (Koren et al., 2017), MaSuRCA v.3.2.8 (Zimin et al., 2013), Flye v.2.3.6 (Kolmogorov et al., 2019) and wtdbg2 (Ruan and Li, 2020). Illumina reads were used to polish the Canu assembly using Nanopolish (Loman et al., 2015), and Pilon v.1.22 (Walker et al., 2014). The completeness of each of the draft genome assemblies was assessed using BUSCO v4 (Simão et al., 2015) using the flowering plant (embryophyte_odb10) orthologous gene data set. Specific commands and flags for each assembly program used are provided in Supplementary File 1.

### Hi-C Scaffolding

*Atriplex hortensis* plants (cv. Golden) were dark-treated for 72 h prior to flash-freezing young leaf tissue in liquid nitrogen. Tissue samples were then shipped to Dovetail Genomics (Scotts Valley, CA, United States) for Chicago® and Hi-C proximity ligation sequencing. Dovetail Chicago® libraries are similar to Hi-C libraries but differ in that they rely on library preparation from *in vitro* rather than *in vivo* reconstituted chromatin that has been cross-linked and subsequently sheared (Moll et al., 2017). Chromosome-scale scaffolds were generated using Dovetail Genomics’ HiRiSE^TM^ assembler.

### Illumina Sequencing and Transcriptome Assembly

The “Golden” variety of *A. hortensis* was grown hydroponically as previously described. Plants were either grown in a control hydroponic solution or in hydroponic solution supplemented with NaCl. For the salt treatment, NaCl was added daily at 50 mM increments to the hydroponic solution of 21-day old plants until a concentration of 350 mM NaCl was reached (7 days). Tissue for RNA extraction was harvested 24 h after 350 mM NaCl concentration was reached. Root, stem and leaf tissue was harvested from both control and treated plants. One-week old whole plantlet and inflorescence (tissue and immature seed) tissues from untreated plants were also collected.

In total, seven libraries were prepared with 180-bp inserts. Sequencing was conducted using the Illumina HiSeq platform at the Beijing Genomics Institute (Shenzhen, China). Reads were trimmed and quality controlled using the program Trimmomatic-0.35 (Bolger et al., 2014). RNA-seq data were aligned to the Hi-C assembly using HiSat v2.2.1 with the max intron length set to 50,000 bp (Kim et al., 2015). Data was then assembled into potential transcripts using StringTie (Pertea et al., 2015) with default parameters.

### Repeat Analysis and Annotation

*Atriplex hortensis*-specific repeats were identified using RepeatModeler v.1.0.11 (Smit and Hubley, 2008–2015). RepeatMasker v.4.0.7 (Smit et al., 2013–2015) was used to classify *A. hortensis*-specific repeats using the RepBase database version 20160829. The MAKER2 v2.31.10 pipeline (Holt and Yandell, 2011) was used to annotate the *A. hortensis* genome with *ab initio* gene predictions using AUGUSTUS (Stanke et al., 2004) species-specific gene models for *A. hortensis*. Additional evidence sources for the annotations included expressed sequence tags (EST) and protein homology from the transcriptomes of *C. quinoa* (Jarvis et al., 2017) and *C. pallidicaule* Aellen (Mangelson et al., 2019) as well as the *A. hortensis* transcriptome produced from the RNA-seq data previously described. The uniprot_sprot database (downloaded 11/13/2018) was used for Basic Local Alignment Search Tool (BLASTp)-based annotation of the gene models.

### Resequencing

Genomic DNA from each of the 21 *A. hortensis* accessions was extracted using the mini-salts protocol reported by Todd and Vodkin (1996). The DNA concentrations and quality were checked using the dsDNA BR Assay from Qubit® 2.0 Fluorimeter. Libraries were sent to Novogene (San Diego, CA, United States) for whole-genome Illumina HiSeq X Ten sequencing (2 × 150-bp paired-end). Reads were trimmed with Trimmomatic using default parameters (Bolger et al., 2014). Reads from each accession were then aligned to the final *A. hortensis* reference genome using Bowtie2 using the very-sensitive-local flag (Langmead and Salzberg, 2012) to produce BAM files that were further marked for PCR duplicates using the MarkDuplicates subroutine in the Picard package.^2^ Single nucleotide polymorphism (SNP) genotype likelihoods and covariances were then determined from the 21 accessions using ANGSD using a *p*-value of 10E-06 for a site being variable (Korneliussen et al., 2014) to produce a genotype likelihood (beagle) file. Multivariate analysis of the covariance data was accomplished using PAST4 (Hammer et al., 2001), while population structure and admixture were then inferred using PCAngsd (Meisner and Albrechtsen, 2018) at *K* = 3 based on the DeltaK method described by Evanno et al. (2005). Bootstrapped (*n* = 1000) UPGMA phylogenies based on Euclidean similarity indices were produced using PAST4 (Hammer et al., 2001).

### Cytogenetics and Genome Size Estimation

*Atriplex hortensis* cv. Golden seeds were germinated on petri dishes for 36 h. Root meristems were collected and immersed in ice water for 24 h. Root meristems were then treated for another 24 h in a 3:1 mixture of ethanol (95%) – glacial acetic acid. Root tips were prepared under a dissecting microscope where they were placed on slides, treated with iron-acetocarmine, warmed on an alcohol burner, and squashed. Chromosomes were examined using a Zeiss Axioplan 2 phase-contrast microscope and images were captured on an Axiocam (Carl Zeiss, Jena, Germany) CCD camera. Fluorescent *in situ* hybridization (FISH) rDNA images of mitotic chromosome preparations of *A. hortensis* cv. “Triple Purple” were taken using yellow-green fluorescing digoxygenin to highlight the NOR-35S region and red fluorescing rhodamine to highlight the 5S region using the protocol described by Maughan et al. (2006). Chromosome spreads and DNA probes for FISH were prepared using the protocol described in Kolano et al. (2012).

Genome-size estimation was conducted using a Beckman Coulter (Miami, FL, United States) Gallios flow cytometer by Agriculture and Agri-Food Canada (AAFC) as described by Yan et al. (2016). Samples were analyzed in triplicate (technical replicates) conducted over three different days. Characteristics of the florescence peaks including mean, nuclei numbers, and coefficients of variation were determined using the R package flowPloidy (Smith et al., 2018). The 2C DNA value of each sample was calculated as: (mean of sample G1peak/mean of standard G1 peak) × 2C DNA content (pg) of the radish (*Raphanus raphanistrum* subsp. *sativus*, estimated 515 Mb) standard.

## Results

### Library Fragmentation

Since Oxford Nanopore Technologies (ONT) sequencing is still relatively new, we tested the relationship between fragmentation strategies, read length and total sequence output to discover the optimal sample preparation method. To achieve sufficient coverage, we developed nine different libraries that were each sequenced independently on different flow cells. In total, the nine libraries yielded 65.4 Gb of data from 5,525,447 reads with a read length N50 of 22,087 bp, a mean read length of 13,487 bp and a mean quality score of nine (Table 2). Individual DNA libraries prepared with fragmentation (Covaris g-TUBEs and ZYMO DNA concentrator-5 column kit) or without fragmentation produced dramatically varied results in terms of read lengths and total sequence yield. Not unexpectedly, the library prepared without fragmentation produced the longest read lengths (N50 = 40,434 bp) but also exhibited the lowest overall sequence yield (1.26 Gb). Fragmentation using the Covaris g-TUBEs at different centrifugation speeds (3,800, 4,000, and 4,200 RPM) produced variable results, but with general trends, specifically: (1) Covaris g-TUBE fragmented libraries always outproduced the non-fragmented library (average yield = 8.67 Gb), but the N50 of the read lengths of these libraries was always smaller (average N50 = 17,166 bp), and (2) lower centrifugation speeds produced longer read lengths (3,800 RPM = 17,979 bp vs 4,200 RPM = 15,939 bp), but with lower yield (3,800 RPM = 8.5 Gb vs 4,200 RPM = 10.9 Gb; Table 2). The two fragmentation libraries produced using the ZYMO DNA kit yielded intermediately to the non-fragmented libraries and the Covaris fragmented libraries, with an average of 3.93 Gb of sequence with a read length N50 of 28,495 bp, suggesting that the ZYMO DNA kit only minimally fragmented the DNA.

**TABLE 2:** Oxford Nanopore library preparation and sequencing statistics. Non-fragmentation and fragmentation techniques were used in sample preparation.

Library	Fragmentation	Total length (Gb)	Totals reads	N50 (bp)	Mean length (bp)	Median length (bp)	Max length (bp)	Mean quality score
1	No Fragmentation	1.26	55,551	40,434	22,617	15,607	194,834	9.1
2	Zymo	5.61	567,514	23,595	9,877	4,522	153,389	9.4
3	Zymo	2.24	133,660	33,394	16,770	9,857	199,575	9.2
4	Covaris, 4,200 RPM	13.04	1,005,270	15,878	11,760	11,017	181,817	8.3
5	Covaris, 3,800 RPM	10.08	854,994	15,277	11,788	11,104	133,274	9.1
6	Covaris, 3,800 RPM	6.94	501,526	20,681	13,760	12,431	164,726	8.9
7	Covaris, 4,200 RPM	8.86	617,385	19,276	14,343	12,932	231,794	9.1
8	Covaris, 4,200 RPM	10.72	1,221,530	12,664	8,778	8,300	149,453	9.1
9	Covaris, 4,000 RPM	6.64	568,017	17,580	11,686	11,115	128,743	8.9
Avg/Total		65.4	5,525,447	22,087	13,487	10,765	170,845	9

### Genome Assembly

**FIGURE 1:**
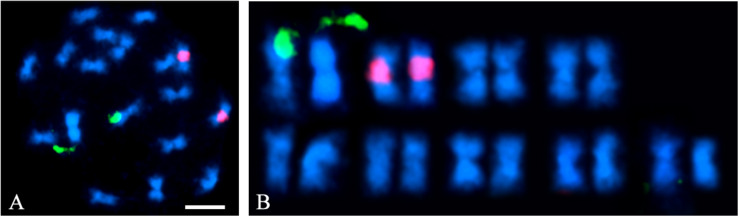
*A. hortensis* chromosome pairs. Nine metacentric chromosome pairs are visible. **(A)** Fluorescent *in situ* hybridization (FISH) using NOR-35S (green) and 5S (red) labeled rDNA probes on mitotic chromosome preparations of *A. hortensis* cv. “Triple Purple.” **(B)** Chromosomes from panel **(A)** arranged as a karyotype. Note the metacentric to submetacentric centromere positions on all nine chromosome pairs.

Flow cytometry indicated that the *A. hortensis* genome is approximately 1.172 Gb (Table 3), while karyotyping of cell nuclei showed that *A. hortensis* carries nine pairs of chromosomes (2*n* = 2*x* = 18). In the *A. hortensis* karyotype, chromosomes were metacentric to slightly submetacentric (Figure 1), and similar in length.

**TABLE 3:** Flow cytometry results of *A. hortensis* (cv. “Golden”) leaf tissue. A C-value of 2.4 picograms yielded a genome size estimate of 1.17 Gb.

Sample	No. technical replicates	Mean 2C value ± S.D. (pg DNA)	Gb per haploid (1C) genome^1^
1	3	2.39 ± 0.0342	1.169 ± 0.017
2	3	2.39 ± 0.0184	1.169 ± 0.009
3	3	2.41 ± 0.0061	1.178 ± 0.003
Average		2.39 ± 0.0196	1.172 ± 0.009

*^1^Haploid genome size was calculated as 1 pg = 978 Mbp per Dolezel et al. (2003).*

Multiple assemblers were tested to determine which would most optimally assemble the *A. hortensis* genome. These assemblers included Canu (Koren et al.), MaSuRCA (Zimin et al., 2013), Flye (Kolmogorov et al., 2019) and wtdbg2 (Ruan and Li, 2020). All assemblers were run with default parameters. The wtdbg2 assembler produced the largest number of contigs and the Flye assembler produced the smallest N50 (Table 4). Both the wtdbg2 and the Flye assemblers produced smaller (total genome size) assemblies relative to the MaSuRCA and Canu assemblers. Both the MaSuRCA and Canu assemblers produced >1 Mb contig N50s, with the Canu assembler producing the least collapsed assembly (relative to the predicted genome size of 1.2 Gb).

**TABLE 4:** Assembly and Benchmarking Universal Single Copy Orthologs (BUSCO) statistics for the MaSuRCA, Flye, wtdbg2, Canu and Hi-C scaffolded Canu assemblies.

Metrics	Assembler				
	MaSuRCA^1^	Flye	wtdbg2	Canu	Canu Hi-C^1^
Number of contigs	2,850	3,009	4,659	3,183	1,325
Total size of the assembly (bp)	914,348,993	813,404,858	867,633,014	964,774,081	965,003,581
Longest (bp)	10,067,120	11,408,532	7,183,448	9,632,068	113,540,806
Shortest (bp)	2,256	178	2,807	1,072	603
Mean (bp)	320,824	270,324	186,227	303,102	728,305
Median (bp)	39,479	100,839	15,813	63,149	35,305
N50 (bp)	1,317,304	698,928	1,176,597	1,114,696	98,884,393
L50	169	282	201	223	5
G+C%	36.84	36.77	36.89	37.06	37.05

	**BUSCO metrics**				

%Complete COGS found [%single, %duplicate]	90.0% [85.8%, 4.2%]	90.6% [88.1%, 2.5%]	86.0% [83.5%, 2.5%]	97.3% [95.1%, 2.2%]^2^	96.7% [95.0%, 1.7%]

*^1^Scaffold statistics are reported for the MaSURca and Canu Hi-C assembly. All other assembly statistics are contig metrics.*

The MaSuRCA assembler uses a hybrid approach for assembly that initially utilizes high-quality short-reads to produce super-reads that are then scaffolded and gap-filled with the long reads to produce high-quality scaffolds that do not require error correction (Zimin et al., 2013). The Flye, Canu and wtdbg2 assemblers are based solely on the error-prone long reads and are thus considered unpolished assemblies and require polishing to correct the inherently high sequencing error rate associated with the ONT technology. We polished the Flye, Canu and wtdbg2 draft assemblies with Nanopolish, which uses the original ONT reads for consensus correction along with two rounds of Pilon, which in turn uses the high-quality Illumina short reads for correction to produce a high-quality, polished set of draft assemblies for comparison (Table 4). We evaluated the final assemblies using Benchmarking Universal Single Copy Orthologs (BUSCO) (Simão et al., 2015) which quantifies gene content completeness based on a large core set of highly conserved orthologous genes (COGs). After polishing, BUSCO was used to identify complete COGs within the various assemblies, which ranged from a low of 86.0% in the Flye assembly, to a high of 97.3% in the Canu assembly. The necessity of the polishing steps was reflected in the increasing BUSCO scores after successive rounds of polishing. For example, the BUSCO scores for complete COGs identified for the original, Nanopolished, Nanopolished+Pilon and Nanopolished+Pilon+Pilon Canu assemblies were 50.5, 73.8, 90.1, and 97.3%, respectively.

Both the MaSuRCA and Canu assemblers produced superior assemblies based on the total size of the contigs, contig N50, and BUSCO scores; however, the polished Canu assembly was ultimately chosen as the draft genome for Hi-C scaffolding due to concerns of repeat collapse within the MaSuRCA assembly as reflected in the smaller total size of the contigs. The polished Canu assembly resulted in 3,183 contigs, spanning 965 Mb, a contig N50 of 1.114 Mb, an L50 of 223, and a BUSCO score of 97.3% (Table 4).

### Chromosome-Scale Scaffolding

To further improve the Canu assembly, contigs were scaffolded using chromatin-contact maps using Dovetail Chicago® and Hi-C libraries. Chicago® library contact maps are based on *in vitro* reconstituted DNA and are ideal for detecting and correcting miss-joins in *de novo* assemblies as well as short-range scaffolding (Putnam et al., 2016). A total of 163 million read pairs (70X coverage) were generated from the Chicago® library and were used to detect misalignments and scaffold the Canu assembly using the HiRiSE^TM^ scaffolder. In total, 429 breaks and 1,421 joins were made, resulting in a net decrease in the total number of scaffolds to 2,191 and a slight decrease in N50 (817 kb) for the assembly. Whenever a join was made between contigs, an “N” gap, consisting of 100 Ns, was created. The total percent of the genome in gaps was less than 0.1%.

The Chicago® -based assembly was then further scaffolded using an *in vivo* Hi-C library created from native chromatin to produce ultra-long-range (10–10,000 kb) mate-pairs. A total of 200 million mate-pair reads, representing a physical coverage of 62×, were generated and scaffolded using the HiRiSE^TM^ scaffolder. In total, 868 joins and no breaks were made, producing a final assembly containing 1,325 scaffolds, spanning a total sequence length of 965 Mb with an N50 and L50 of 98.9 Mb and 5 scaffolds, respectively. Nine chromosome-scale scaffolds were assembled containing 94.7% of the total sequence length. The chromosome-scale scaffolds ranged in size from 93.6 to 113.5 Mb and were numbered sequentially based on scaffold length (e.g., Ah1–Ah9). Scaffold joins produced by the Hi-C mate-pairs introduced new “N” gaps in the assembly, thereby increasing the number of gaps in the assembly to 2,295. The final number of “N” nucleotides in the final Hi-C assembly was 229,050 (<0.1%; Table 2).

A BUSCO analysis of the final Hi-C assembly identified 1,331 (96.8%) complete COGs from the *Embryophyta* database (*n* = 1375), of which only 1.7% (23) were duplicated – reflecting the diploid nature of the *Atriplex* genome and suggesting that only minor paralogous duplications have occurred. Another nine (0.7%) fragmented COGs were identified. Only 35 COGs were missing, which is indicative of a highly complete assembly.

### Repeat Features

The RepeatModeler and RepeatMasker pipelines were used to annotate and mask the repeat fraction of the Hi-C assembly. Approximately 66% (639.6 Mb) of the genome was annotated as repetitive, which is slightly higher than the repetitive fraction classified for other members of the *Amaranthaceae/Chenopodiaceae* alliance with reference genomes [48% in *Amaranthus hypochondriacus* L. (Lightfoot et al., 2017), 64% in *Spinacia oleracea* (Li et al., 2019), 63% in *Beta vulgaris* L. (Flavell et al., 1974), 64.5% in *C. quinoa* (Jarvis et al., 2017)]. The most common repeat elements identified were long-terminal repeat retrotransposons (LTR-RT). The LTR-RTs are the most abundant genomic component in flowering plants (Du et al., 2010; Galindo-Gonzalez et al., 2017) and their frequency is strongly correlated with increased genome size (Michael, 2014). Of the various LTR-RTs present in the *A. hortensis* genome, *Gypsy*-like (31.90%) and *Copia*-like (11.13%) elements represent greater than 40% of the genome and are in a 3:1 (*Gypsy*:*copia*) ratio, similar to the 2.9:1 ratio reported for 50 sequenced plant genomes (Ou and Jiang, 2018). An additional 5.14% (49.5 Mb) of the genome was classified as low-complexity (satellites, simple repeats, and rRNAs), while 14.86% (143 Mb) of the genome was characterized as unclassified repetitive elements (Table 5) – presumably representing *Atriplex*-specific repeat elements that will undoubtedly be important for understanding the evolution of the *A. hortensis* genome.

**TABLE 5:** Repetitive element classification for final assembly (Canu Hi-C) as reported by RepeatMasker.

Repeat class^*a*^	Repeat name	Count	bp masked	Masked (%)
DNA		2,937	2,206,004	0.23
	CMC-EnSpm	24,140	13,861,122	1.44
	Crypton	972	83,799	0.01
	MULE-MuDR	13,906	6,550,989	0.68
	MuLE-MuDR	2,306	1,334,054	0.14
	PIF-Harbinger	1,772	892,861	0.09
	TcMar-Mogwai	1,261	652,191	0.07
	TcMar-Stowaway	32,881	5,565,216	0.58
	hAT	548	112,059	0.01
	hAT-Ac	10,241	2,812,539	0.29
	hAT-Tag1	1,772	308,038	0.03
	hAT-Tip100	6,258	7,096,629	0.74
LINE				
	CRE-II	447	335,625	0.03
	Jockey	2,157	567,406	0.06
	L1	8,788	7,141,529	0.74
	L2	14,243	21,727,481	2.25
	Penelope	2,431	992,653	0.10
	R1	566	220,850	0.02
	RTE-BovB	6,587	2,055,784	0.21
LTR		7,950	1,885,731	0.20
	Caulimovirus	351	589,622	0.06
	Copia	62,884	107,371,058	11.13
	DIRS	4591	1,952,514	0.20
	Gypsy	167,590	307,636,295	31.9
	Pao	76	7,473	0.00
	Caulimovirus	7,950	1,885,731	0.20
RC				
	Helitron	6,262	2,307,357	0.24
SINE				
	tRNA	418	50,479	0.01
Unknown		451,250	143,307,912	14.86
Total interspersed		835,585	639,625,270	66.33
Low_complexity		31,237	1,603,682	0.17
Satellite		128	11,962	0.00
Simple repeat^*b*^		216,414	47,610,726	4.94
rRNA		598	309,846	0.03
Total		1,083,962	6,89,161,486	71.46

*^*a*^LINE, long interspersed nuclear elements; LTR, long terminal repeat; RC, Rolling circle. ^*b*^The most common mono- di-, tri-, and tetra- nucleotide repeat motifs were (T)n, (TA)n, (ATT)n, (TTTA)n, respectively.*

A BLAST search (Altschul et al., 1990) of the complete rRNA gene sequence found in *C. quinoa* (DQ187960.1) was conducted to identify the 35S rRNA gene (NOR) location in the *A. hortensis* genome using the *C. quinoa* sequence as query. The 35S rRNA locus was located on chromosome Ah6. Another BLAST search was conducted to identify matches for the 5S rRNA gene locus in *A. hortensis*, again using the homologous 5S rDNA repeat sequence in *C. quinoa* (DQ187967.1) as the query. The 5S rDNA sequences mapped primarily to chromosome Ah4 and to several other smaller unscaffolded contigs that did not assemble into specific chromosomes. The appearance of these smaller scaffolds in the BLAST search results was not surprising as 5S rDNA repeats are highly repetitive and of low-complexity, and thus extremely difficult to assemble and scaffold accurately. A FISH analysis of mitotic chromosome preparations for *A. hortensis* cv. Triple Purple revealed a physical location of a single NOR-35S (green) locus and of a single 5S (red) rRNA gene tandem repeat-array locus (Figure 1). The identification of the cytological and genomic position of the 5S rRNA and 35S rRNA gene loci gives unique identities to two of the nine chromosome pairs in the *A. hortensis* kayotype (specifically Ah4 and Ah6).

**FIGURE 2:**
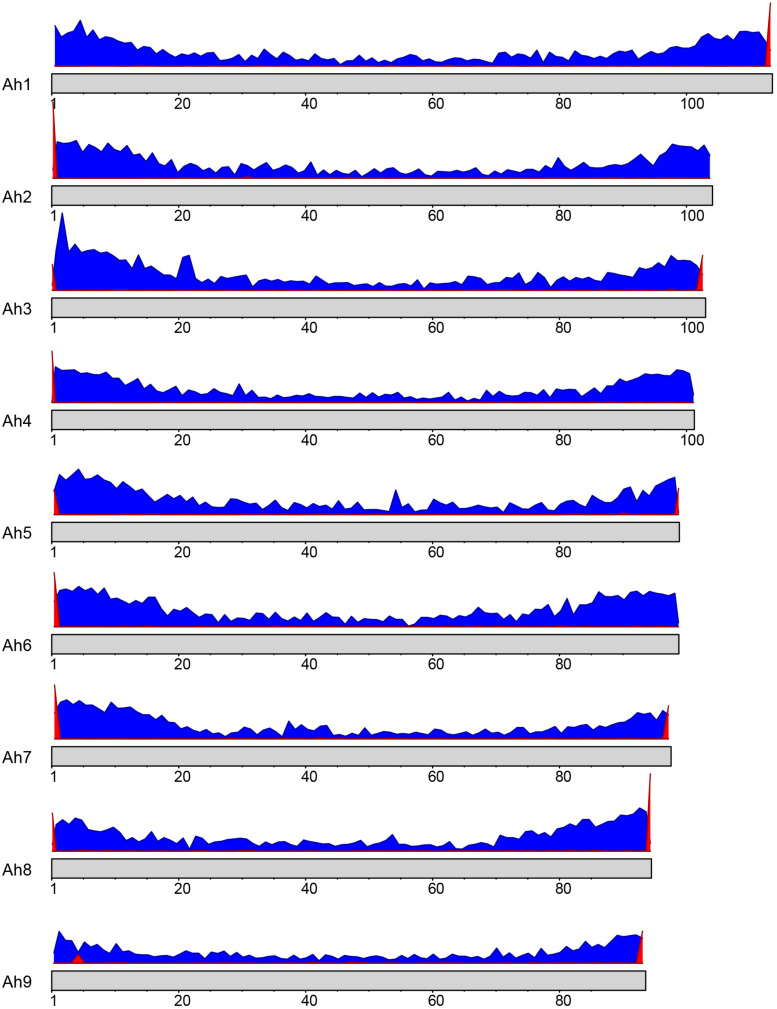
Gene density (blue) and telomere positioning (red) on the *A. hortensis* chromosomes. A conserved telomere repeat sequence in plants (TTTAGGG; Richards and Ausubel, 1988) was used to locate telomere positions using BLAST searches. The *x-*axis represents the position on the chromosome, while the *y-*axis represents the frequency of genes or telomeric repeats in each bin.

The sequence for telomeric repeats in plants is highly conserved and has been identified as TTTAGGG (Richards and Ausubel, 1988). A BLAST search of this sequence motif against the nine *A. hortensis* chromosomes identified tandemly repeated telomeric sequences on at least one end of each of the nine chromosome assemblies with a total of 13 telomere-like repetitive regions identified (Figure 2). Four of the nine chromosomes had telomere-to-telomere assemblies (telomeres identified on both ends of the chromosome assembly).

### Genome Annotations

**FIGURE 3:**
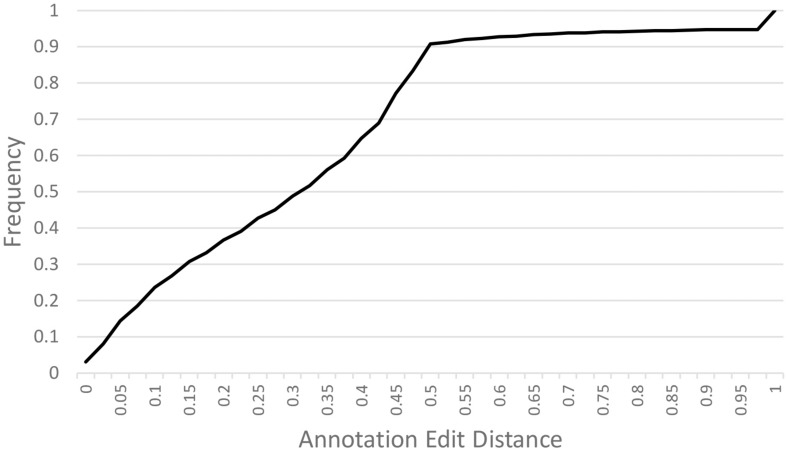
Annotation Edit Distance (AED) calculated for MAKER predicted gene models. Annotation Edit Distance (AED) is used to measure the quality of a genome annotation. This is calculated by combining annotation values corresponding to specificity and sensitivity. AED values of 0.50 and below are good annotations and values of 0.30 and below are high-quality annotations (Holt and Yandell, 2011).

A *de novo A. hortensis* transcriptome, derived from 30–40 million RNA-seq reads each from stem, leaf, floral and whole plantlet tissues, consisted of 272,255 isoforms with an N_50_ of 3,325 bp and a mean length of 1,956 bp. The *A. hortensis* transcriptome, along with the EST and peptide models from *C. quinoa* and *C. pallidicuale* and the uniprot-sprot database, were provided as primary evidence for annotation in the MAKER pipeline. The RNA-seq data mapped with high efficiency to the final genome assembly, with an overall alignment rate of 92% and with 81.5% of the pair reads aligning concordantly exactly one time, with only 4.26% aligning more than once concordantly – suggestive of a high-quality genome assembly and reflective of the diploid nature of the *A. hortensis* genome. The MAKER pipeline identified a total of 39,540 gene models and 2,555 tRNA genes. The average length of genes identified by MAKER was 1,750 bp. The completeness of the annotation was assessed by BUSCO which identified 1,278 (92.9%) complete COGs from the transcript annotation (Complete: 92.9% [Single Copy: 90.4%, Duplicated: 2.5%], Fragmented: 4.7%, Missing: 2.4%). To assess the quality of the annotations, we used the mean Annotation Edit Distance (AED) which is calculated by combining annotation values corresponding to specificity and sensitivity. AED values of 0.5 and below are considered good annotations, and values of 0.30 and below are considered high quality annotations (Holt and Yandell, 2011). Over 90% of the genome models have an AED value <0.5, with the majority (51.7%) of the models having AED values below 0.325 (Figure 3). An analysis of the completeness of the gene models was further assessed by comparing the matched length of the transcripts with orthologous *C. quinoa* transcripts. Orthologs were determined using BLAST analysis (*e*-value < 1e-20) with the max target set to 1. Of the 18,657 orthologs identified with *C. quinoa*, ∼80% (14,764) covered at least 70% of the *C. quinoa* orthologs. The AED score coupled with the BUSCO assessment and ortholog analysis are suggestive of a high-quality genome assembly and annotation. In addition, the observed chromosomal distribution of the annotated genes, with higher gene density near the ends of chromosomes and lower gene density in the centromeric regions (Figure 2), is suggestive of a high-quality genome assembly and annotation. An examination of the self-synteny map (not shown but accessible via CoGe) revealed no obvious blocks of paralogous genes.

### Genomic Comparison and Features

Several species within the *Amaranthaceae/Chenopodiaceae* alliance have chromosome-scale genome assemblies, including the ancient allotetraploids *C. quinoa* (Jarvis et al., 2017) (2*n* = 4*x* = 18) and *Amaranthus hypochondriacus* (Lightfoot et al., 2017) (2*n* = 4*x* = 16), and the diploid *Beta vulgaris* (Funk et al., 2018) (2*n* = 2*x* = 9). Previous phylogenic research using chloroplast DNA (*rbcL* gene, *atpB-rbcL* spacer) and nuclear rDNA internal transcribed spacer (ITS), clearly demonstrated that *Atriplex* is more closely related to *Chenopodium* and *Beta*, which are both found in the same family *Chenopodiaceae* s. str. but in different subfamilies, *Chenopodioideae* (*Chenopodium* and *Atriplex*) and *Betoideae* (*Beta*), while *Amaranthus* is more distantly related to these chenopods and is found within the family *Amaranthaceae* s. str., subfamily *Amaranthoideae* (Kadereit et al., 2003, 2010; Fuentes-Bazan et al., 2012; Morales-Briones et al., 2020). Syntenic relationships between *A. hortensis* and these other genomes were explored using DAGChainer (Haas et al., 2004), which identifies syntenic blocks of collinear homologous gene pairs between genomes.

**FIGURE 4:**
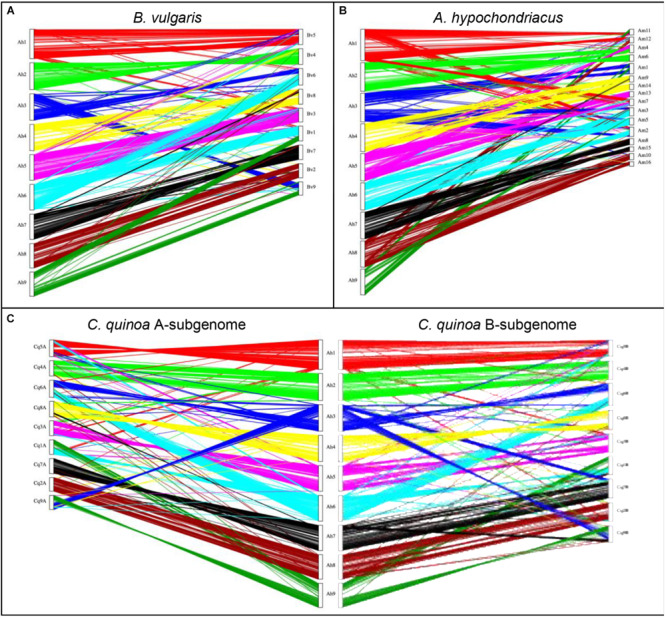
Genomic comparison of *A. hortensis* with *B. vulgaris*, *A. hypochondriacus*, and *C. quinoa*. The dual synteny plots show syntenic regions between *A. hortensis* and *B. vulgaris* **(A)**, *A. hypochondriacus* **(B)** and *C. quinoa* **(C)** coding sequences. The dual synteny plot of the *C. quinoa* genome is divided into A- and B-subgenomes with *A. hortensis* in the center.

A synteny analysis of *A. hortensis* and *B. vulgaris* identified 226 shared syntenic blocks between the genomes, with 11,697 colinear gene pairs (averaging 52 gene pairs/block) spanning 469 and 616 Mb of the *B. vulgaris* and *A. hortensis* genomes, respectively. Moreover, syntenic block sizes between the species were also correlated (R^2^ = 0.36), further reflecting a shared ancestry of two species. One-to-one orthologous relationships between *A. hortensis* and *B. vulgaris* chromosomes (Figure 4 and Table 6) were clearly ascertained for six of the nine *A. hortensis* chromosomes: Ah1 = Bv5 (100% shared syntenic block sequence), Ah2 = Bv4 (99%); Ah4 = Bv8 (100%); Ah5 = Bv3 (100%); Ah7 = Bv7 (100%); Ah8 = Bv2 (100%). The remaining three *A. hortensis* chromosomes shared substantial levels of synteny with multiple *B. vulgaris* chromosomes, suggestive of intergenomic rearrangements (i.e., reciprocal translocations), with Ah3 = Bv6 (51%), Bv9 (49%); Ah6 = Bv6 (52%), Bv1 (48%); and Ah9 = Bv9 (54%), Bv1 (44%). We note that we cannot exclude that these rearrangements are possible misassembles – although our Hi-C data strongly supports the current placements.

**TABLE 6:** Orthologous genes were identified between *A. hortensis* and beet (A), *C. quinoa* (B) and *Am. hypochondriacus* (C) to detect orthologous chromosome relationships.

	Ah1	Ah2	Ah3	Ah4	Ah5	Ah6	Ah7	Ah8	Ah9	Total synteny spanned (bp)
**(A)**										
Bv5	75,985,214									75,985,214
Bv4		76,231,045		205,876						76,436,921
Bv6			32,474,644			38,024,142			1,018,214	71,517,000
Bv8		1,043,269		59,005,657						60,048,926
Bv3					76,200,679					76,200,679
Bv1						35,497,946			24,584,006	60,081,952
Bv7							68,960,315			68,960,315
Bv2								65,798,714		65,798,714
Bv9			31,029,170						29,976,099	61,005,269
**(B)**										
Cq5A	88,356,818		80,733							88,437,551
Cq5B	85,221,237									85,221,237
Cq4A		78,889,108						678,948		79,568,056
Cq4B		74,788,751		265,848						75,054,599
Cq6A			34,880,745			45,906,343				80,787,088
Cq6B			30,549,711			43,708,464				74,258,175
Cq8A		844,472		63,922,236	73,334					64,840,042
Cq8B		844,472		68,256,659						69,101,131
Cq3A					72,931,389	1,972,202				74,903,591
Cq3B					73,061,839					73,061,839
Cq1A						39,186,132			24,859,805	64,045,937
Cq1B						32,666,424		3,006,287	29,799,350	65,472,061
Cq7A							68,938,800			68,938,800
Cq7B			12,915,209				60,375,606	2,102,719		75,393,534
Cq2A								68,395,255		68,395,255
Cq2B		1,684,254		794,038		7,716,047		57,060,920		67,255,259
Cq9A			34,390,946						29,902,118	64,293,064
Cq9B			21,296,635				10,957,411		27,531,165	59,785,211
**(C)**										
Am11	34,077,858					1,414,077		5,216,151	7,553,686	48,261,772
Am12	31,526,741				604,945	329,285		9,294,151		41,755,122
Am4		41,122,991			30,756,424					71,879,415
Am6		46,411,045								46,411,045
Am1			47,400,936						31,083,078	78,484,014
Am9		609,018		45,747,049			521,887			46,877,954
Am14				41,664,113						41,664,113
Am13					44,814,461					44,814,461
Am7	6,842,070				17,248,169	1,330,917				25,421,156
Am3	1,167,442		24,822,048			25,821,038				51,810,528
Am5						25,008,790			12,043,145	37,051,935
Am2	23,542,392	1,607,269	15,871,055			16,950,212				57,970,928
Am8							55,245,256			55,245,256
Am15							40,042,725			40,042,725
Am10	1,585,265		54,032		144,414			37233979		39,017,690
Am16						283,173		16935472		17,218,645

*Total syntenic bases are shown between all chromosome comparisons. Syntenic relationships are colored red and transition to white as the amount of synteny decreases.*

*Atriplex hortensis* and *B. vulgaris* are both diploid and share a haploid chromosome number (*n* = 9), whereas *C. quinoa* is an allotetraploid member (showing amphidiploid inheritance) of the subfamily *Chenopodioideae*, having experienced an ancient allopolyploidization event (Storchova et al., 2015). Our analysis of synteny between *C. quinoa* and *A. hortensis* identified a combined total of 24,710 syntenic gene pairs, spanning 1.1 Gb and 1.3 Gb of the *C. quinoa* and *A. hortensis* genome, respectively, using a tetraploid-to-diploid (2:1) analysis. The synteny observed among the *A. hortensis* and *C. quinoa* chromosomes suggests several orthologous relationships with known homeologous *C. quinoa* chromosome pairs, including Ah1 = Cq5A (51% shared syntenic block sequence), Cq5B (49%); Ah2 = Cq4A (50%), Cq4B (48%); Ah4 = Cq8A (48%), Cq8B (51%); Ah5 = Cq3A (50%), Cq3B (50%); Ah7 = Cq7A (49%), Cq7B (43%); Ah8 = Cq2A (52%), Cq2B (43%). As with *B. vulgaris*, *A. hortensis* chromosomes Ah3, Ah6 and Ah9 have large rearrangements showing synteny to Cq1A&B, Cq6A&B and Cq9A&B.

**FIGURE 5:**
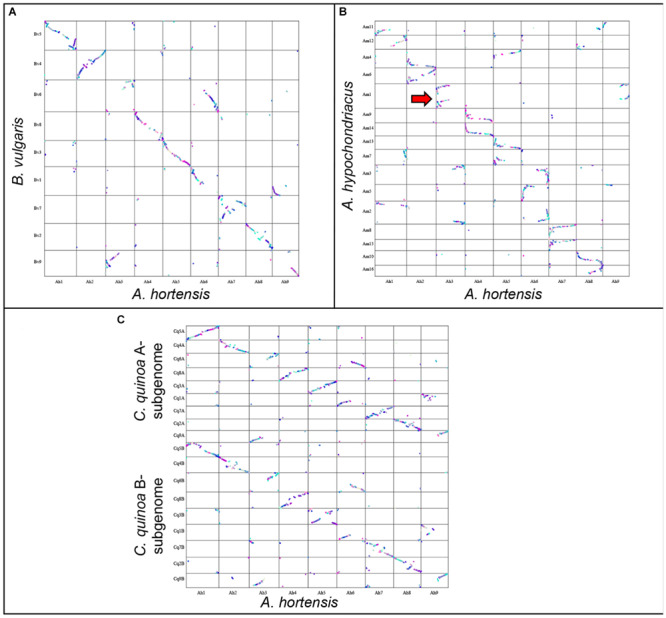
Genomic comparison of *A. hortensis* with *B. vulgaris*, *Am. hypochondriacus* and *C. quinoa*. Synteny dotplot showing syntenic coding sequences between *A. hortensis* and *B. vulgaris* **(A)**, *Am. hypochondriacus* **(B)** and *C. quinoa* **(C)** coding sequences. Increasing color intensity is associated with increasing homology.

*Atriplex hortensis*, *B. vulgaris* and *C. quinoa* share a base chromosome number of *x* = 9, whereas the base number in *Amaranthus* is *x* = 8, due to a chromosome loss (Am5) and a chromosome fusion (Am1; Lightfoot et al., 2017). The amaranths belong to the family *Amaranthaceae* s. str. Subfamily *Amaranthoideae* and were thus expected to be the most divergent of the three genomes compared. Indeed, while our genome comparison of *A. hortensis* with *A. hypochondriacus* clearly showed synteny (Figure 4 and Table 6), the size of the 410 syntenic blocks (12,306 syntenic gene pairs) observed was the smallest of the three genomes (Bv: 2.1 Mb/block; Cq: 2.7 Mb/block; Am: 0.84 Mb/block), accompanied by the lowest block size correlation between the species (*Bv*: R^2^ = 0.36; *Cq*: R^2^ = 0.42; *Am*: R^2^ = 0.04). These decreases are reflective of the more distant evolutionary relationship between *Atriplex* and *Amaranthus* within the family. We confirm the chromosome fusion event in *Amaranthus* as seen by the synteny plot of Ah3, where Ah3 aligns twice with Am1 (Figure 5; red arrow). Although many additional rearrangements are present which obscure one-to-one orthologous chromosome relationships with the known homeologous amaranth chromosomes (Lightfoot et al., 2017), several can be confirmed: Ah2 = Am4 (46%), Am6 (52%); Ah4 = Am9 (52%), Am14 (48%); Ah7 = Am8 (58%), Am15 (42%; Table 6).

**FIGURE 6:**
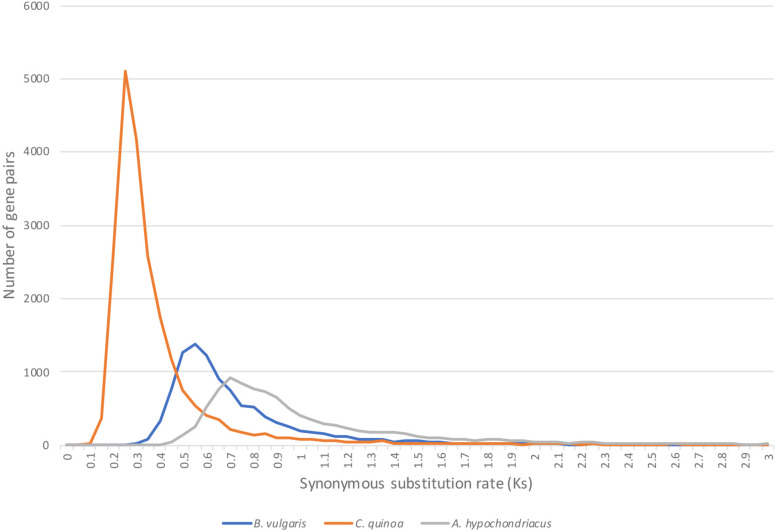
The age of the evolutionary split between *A. hortensis* and *B. vulgaris*, *C. quinoa*, and *A. hypochondriacus* was estimated using the rate of synonymous substitutions per synonymous site (Ks) calculated from othologous gene pairs. *C. quinoa* is most closely related to *A. hortensis*, with a clear peak present at Ks = 0.25, followed by *B. vulgaris* (Ks peak = 0.55), while *A. hypochondriacus* is more distantly related, with a Ks peak = 0.7.

To elucidate the timing of the evolutionary events that separate *Atriplex* from *C. quinoa*, *B. vulgaris* and *A. hypochondriacus*, we calculated the rate of synonymous substitutions per synonymous site (K_s_) in duplicate gene-pairs between the species (Figure 6) using the CodeML (Yang, 2007) tool on the CoGe platform (genomevolution.org/coge). As expected, *C. quinoa* is most closely related to *A. hortensis*, with a clear peak present at Ks = 0.25, followed by *B. vulgaris* (Ks peak = 0.55), while *A. hypochondriacus*, as expected, is more distantly related, with a Ks peak = 0.7. The timing of the divergence events (time to last common ancestor) can be established using the Ks peak values and synonymous mutation rates, such as the core eukaryotic rate (8.1E–09) proposed by Lynch and Conery (2000) or with lineage specific rates, calibrated to the fossil record. Kadereit et al. (2003) used three paleobotanical fossils to establish a much lower synonymous substitution rate for *Chenopodioideae* (2.8–4.1E-09), which showed rate constancy among the lineages studied, suggesting that the *Amaranthaceae-Chenopodiaceae* have a lower nucleotide substitution rate than other angiosperms, including the *Arabidopsis* rate (1.5E-08). The CodeML workflow in CoGe identifies syntenic gene pairs between species, extracts coding sequences, and aligns protein sequences using the Needleman–Wunsch alignment algorithm, which is then back-translated to a codon alignment that is then used for Ks estimation. Using the lower substitution rates calculated by Kadereit et al. (2003), we date the last shared common ancestor between *A. hortensis* and *C. quinoa*, *B. vulgaris*, and *A. hypochondriacus* to approximately 30.4 – 44.6 MYA, 67.1 – 98.2 MYA, and 85.3 –125 MYA, respectively.

### Resequencing

**FIGURE 7:**
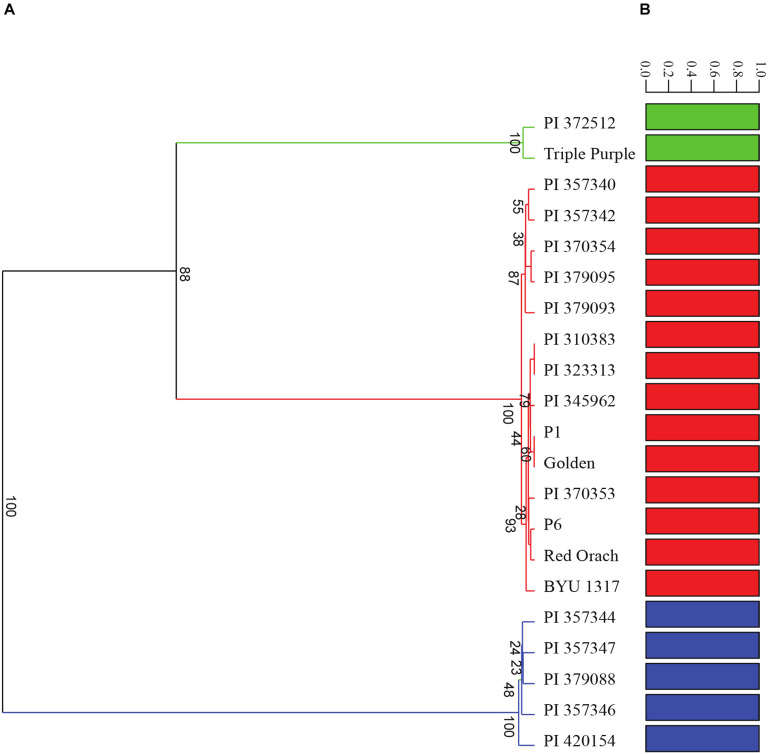
Analysis of the diversity panel using **(A)** 100-bootstrap UPGMA tree based on a Euclidean distance similarity index and **(B)** admixture analysis using genotype posterior probabilities for 17,711,684 SNPs. Accession numbers correspond with those found in Table 1.

A diversity panel consisting of 21 diverse accessions of *A. hortensis* (Table 1) was re-sequenced using Illumina paired-end sequencing, resulting in an average of 13X coverage (13.2 Gb) per accession. Following alignment and genotype likelihood calling with ANGSD (Korneliussen et al., 2014), a total 17,711,684 SNPs were filtered from the 846,491,542 sites analyzed using a 5% minimum minor allele frequency. A principal components analysis of the covariance data using PC1 and PC2 explained a total of 99.92% of the total variation and clearly identified three clusters of *Atriplex* accessions, which also agreed with our DeltaK analysis of the number of groups in the data set (*K* = 3; Figure 7B). Analysis of the 1000-bootstrap, consensus tree identified three distinct clades, with two accessions including the commercial cultivar Triple Purple and a wild accession collected in Alberta, Canada forming the first clade. The second clade consisted of four cultivated accessions of Serbia/Montenegro origin with a single wild accession at their root originating from France. The last and largest clade consisted of two subgroups with the first subgroup consisting of five cultivated lines from Serbia/Montenegro and a second subgroup consisting of four commercially available cultivars (obtained from Wild Garden Seed and Baker Creek Heirloom Seeds) and four accessions from disparate localities across Europe (Poland, Uzbekistan, Norway and Serbia) that were rooted by a wild accession collected in Utah, United States (Figure 7A). The structure plots (Figure 7B) indicate little to no admixture among the subpopulations, suggesting three distinct subpopulations with little to no interbreeding.

## Discussion

Multiple different libraries were prepared for ONT sequencing, including with and without fragmentation, to ascertain the influence of fragmentation on sequencing yield and read length – both important components of successful genome assembly. Fragmentation consistently improved throughput and yield, with the Covaris g-TUBEs producing the most effective and least variable fragmentation (i.e., based on sequencing yield and read length variation). The effect of centrifugation speed (3,800, 4,000, and 4,200 rpm) was also an important, albeit less controllable, factor. In general, higher centrifugation speeds produced higher yields, but concomitantly with decreased read lengths. Indeed, flow cell nanopores remained active for longer periods with fragmented libraries as compared to those without fragmentation. Kubota et al. (2019) demonstrated a similar correlation between DNA length and nanopore inactivity, with inactivity increasing exponentially in relation to increasing DNA molecule size. Nivala et al. (2013) suggested that one possible reason for this could be that longer molecules correlate with an increased presence of secondary and/or tertiary structures in the DNA molecules. Nanopores are restricted to the width of one DNA molecule at a time; thus, if secondary and/or tertiary structures are present in the DNA molecules, they increase the probability of clogging the nanopores, rendering them inactive. The combination of Covaris g-TUBE libraries prepared with differing centrifugation speeds resulted in a dataset with enough yield to provide ample coverage to compensate for the high error rate of ONT sequencing while still yielding long reads needed to span repetitive or otherwise problematic genomic regions (Table 2).

Canu (Koren et al., 2017), MaSuRCA (Zimin et al., 2013), Flye (Kolmogorov et al., 2019) and wtdbg2 (Ruan and Li, 2020) assemblers were used to assemble the ONT sequence data to ascertain which assembly program would perform best with the *A. hortensis* ONT sequence data. There were substantial differences in the overall time to finish an assembly, with wtdbg2 being the fastest of the assemblers tested. However, the MaSuRCA and Canu assemblies produced superior assemblies in terms of total contig size, N50, L50 and BUSCO statistics (Figure 2), with the polished Canu assembly ultimately being chosen as the draft genome for Hi-C scaffolding due to concerns of repeat collapse within the MaSuRCA assembly as reflected in its smaller total size of contigs assembled – a concern noted by Kolmogorov et al. (2019) who demonstrated the difficulty of assembling telomeric and centromeric chromosome regions with the MaSuRCA assembler. Three rounds of polishing were conducted utilizing Nanopolish followed by two rounds of Illumina read-based Pilon correction. Nanopolish uses an index to detect misassemblies based on sequencing-generated signal levels generated from the original nanopore sequence data that correspond to likelihood ratios, while Pilon uses read alignments of high-quality Illumina reads to consensus-correct the draft genome (Walker et al., 2014; Loman et al., 2015). RACON (Vaser et al., 2017), another popular long-read consensus polisher that can use ONT and Illumina sequence for consensus correction, was also tested as a substitute for both Nanopolish and Pilon but showed no significant enhancement to the final BUSCO statistics (data not shown). We note that over-polishing an assembly can also be problematic, as seen by a decrease in BUSCO scores, and should therefore be avoided. In our assembly, a third round of polishing did not improve BUSCO scores.

Unsurprisingly, the B subgenome of *C. quinoa*, which is approximately 25% larger than the assembled A subgenome, shared more and longer syntenic blocks (209 vs 189; 2.9 vs 2.4 Mb average) with *A. hortensis*. The higher synteny with the B subgenome of *C. quinoa* may also reflect a closer ancestry of *A. hortensis* with the B subgenome – whose closest extant known *Chenopodium* species (*C. suecicum* Murr or *C. ficifolium* Sm.) are of Old-World origin, similar to that of *A. hortensis*. We note that the A-subgenome of *C. quinoa* is suspected to be of New World origin with its closest known extant species being *C. watsonii* A. Nelson, which is native to the southwestern United States (Jellen et al., 2019). It should, however, be noted that at least one diploid with the A-genome, *C. bryoniifolium* Bunge, is native to eastern Siberia (Walsh et al., 2015; Mandák et al., 2018a). Moreover, a new allohexaploid species containing the A-subgenome from *C. bryoniifolium* has been recently described from the Far East of Russia as *C.* luteorubrum Mandák & Lomonosova (Mandák et al., 2018b). Thus, an East Asian origin of the A-genome lineage in *Chenopodium*, with its subsequent trans-Beringian migration and explosive diversification in the Americas, cannot be excluded at the present state of our knowledge; however, that scenario is less parsimonious than the New World origin of the A-genome lineage.

The genome of *A. hortensis* is highly repetitive with approximately 66.3% of the sequence containing interspersed repetitive sequence. By comparison, the genome of quinoa is 64.5% repetitive (Jarvis et al., 2017). Genomes that contain substantial repeat fractions can be difficult to assemble correctly. To overcome this challenge, Hi-C chromosome-contact maps were used for genome scaffolding which dramatically increased the continuity of the assembly, producing nine chromosome-sized scaffolds presumably representing each of the haploid chromosomes in *A. hortensis* (*n* = 9). Additionally, the Hi-C chromatin contact maps leverage the spatial orientation of the chromatin to identify and correct misassemblies in the overlap-layout-consensus assembly produced by Canu that potentially would have gone unnoticed. The nine chromosome pairs in *A. hortensis* are metacentric to slightly submetacentric (Figure 1). Due to the difficulty in assembling highly conserved and repetitive sequence regions within telomeres, the identification of 13 of the possible 18 telomeric ends is indicative of a highly complete, chromosome-scale genome assembly (Figure 2). The unexpected location of telomeric sequences in the subtelomeric region of one of the arms of chromosome Ah5 could reflect a potential assembly error – although careful inspection of the chromatin maps for this region do not show any indications of misassembly. Similar paracentric inversions have been seen in other species which result in telomere-specific tandem repeats being present in abnormal locations in plant chromosomes (Tek and Jiang, 2004). Nonetheless, additional research, potentially including optical mapping (e.g., BioNano genomics) and/or high-density linkage map development (neither of which have been developed for *A. hortensis*) should be targeted to this region to verify the orientation of this segment of the chromosome. Such investigations will also help verify the assemblies of chromosomes Ah3, Ah6, and Ah9, which show syntenic relationships with multiple *B. vulgaris* and *C. quinoa* chromosomes arms, thus obscuring their orthologous relationships. Such research would undoubtedly provide additional insight into the chromosomal evolution that characterizes the family *Chenopodiaceae* s. str. and the whole *Amaranthaceae*/*Chenopodiaceae* alliance – such as the homoelog loss and chromosomal fusion reported in *Amaranthus hypochondriacus* (Lightfoot et al., 2017).

It is not surprising that the North American-derived materials grouped with European accessions, as it is commonly understood that the center of origin of *A. hortensis* is the Trans-Himalayan (central Asia and Siberia) and Southeast European regions and that it was likely introduced during the third century B.C. into the Mediterranean littoral and from there to the Americas in Colonial times (Ruas et al., 2001). The species has become locally naturalized along riverbanks, roadsides, and ditches in parts of the Great Basin of North America (personal observations). There is also evidence of its use as a food in Switzerland as early as the Neolithic Age (Andrews, 1948), suggesting widespread, albeit ancient use of the species. Unfortunately, the United States National Plant Germplasm System curates only 45 *A. hortensis* accessions, of which very few are publicly available. The identification of three highly distinct clades, showing only limited admixture in our results, emphasizes the need for additional collections of wild and cultivated germplasm from throughout its native range, particularly in its presumed center of origin. Indeed, of the 45 curated accessions at the USDA, nearly two-thirds (28) are derived from a single European region corresponding to the Balkan Peninsula in Southeast Europe (Serbia-Macedonia). Subsequent phylogenetic analysis using materials from much broader geographic collections should improve our understanding of extant genetic variation and speciation processes within *A. hortensis*.

## Data Availability Statement

The raw sequences are deposited in the National Center for Biotechnology Information (NCBI) Sequence Read Archive database under the BioProject ID PRJNA607334 with the following sequence read archive (SRR) accession numbers: SRR11147376 (Nanopore data), SRR11147367 – SRR11147368 (Hi-C), SRR11147369 – SRR11147375 (transcriptome) and SRR11123164 – SRR11123184 (resequencing panel; Table 1). Bulk data downloads, including annotations and BLAST analysis, and JBrowse viewing of the final Hi-C assembly are available at CoGe (https://genomevolution.org/coge/;Genomeid56906). The scaffold names in CoGe corresponding to specific pseudo chromosome assemblies are as follows: Scaffold_552_HRSCAF_710 = Ah1; Scaffold_579_HRSCAF_742 = Ah2; Scaffold_1312_HRSCAF _2063 = Ah3; Scaffold_481_HRSCAF_623 = Ah4; Scaffold_390 _HRSCAF_510 = Ah5; Scaffold_1281_HRSCAF_1836 = Ah6; Scaffold_1313_HRSCAF_2064 = Ah7; Scaffold_1311_HRSCAF _2062 = Ah8; Scaffold_291_HRSCAF_384 = Ah9. In addition, the variant call file (VCF) for the diversity panel is available as a download from CoGe (ID# 15277).

## Author Contributions

PM, ENJ, and EWJ conceived and designed the study. SH and DL performed the sequencing experiments and managed the plant material. SM performed the flow cytometry experiments, and provided plant material, taxonomic information, and phylogenetic analysis. EWJ provided the assembly bioinformatics and expertise. BK preformed the fluorescent in situ hybridization experiments. PM, SH, and ENJ wrote the manuscript. All authors read and approved the final manuscript.

## Conflict of Interest

The authors declare that this study received funding from General Mills, Inc. The funder had provided financial assistance based on an initial interest in investigating development of new specialty crops. The authors declare that the funder was not involved in the study design, collection, analysis, interpretation of data, the writing of this article or the decision to submit it for publication. EJ was initially employed by General Mills and subsequently left to form 25:2 Solutions. The remaining authors declare that the research was conducted in the absence of any commercial or financial relationships that could be construed as a potential conflict of interest.

## Footnotes

1 https://npgsweb.ars-grin.gov/

2 http://broadinstitute.github.io/picard/

## Abbreviations

BLAST: basic local alignment search tool
Mbp: megabases
MYA: million years ago
ONT: Oxford nanopore technology
SNP: single nucleotide polymorphisms.
