# Supplementary material for: A Chromosome-Scale Assembly of the Garden Orach (Atriplex hortensis L.) Genome Using Oxford Nanopore Sequencing
Source: Front Plant Sci. 2020 May 25;11:624. doi: 10.3389/fpls.2020.00624 (PMC7261831; doi:10.3389/fpls.2020.00624)
Supplement: Supplementary file 1 [file Data_Sheet_1.PDF]

Supplemental Information – Assembly commands

Canu

```
canu -d atriplex -p atriplex_60 genomeSize=1100m maxMemory=500g maxThreads=24 corMhapSensitivity=normal
corOutCoverage=40 \
merylMemory=500g merylThreads=24 ovsMethod=parallel \
gridOptions="--qos=pws --time=72:00:00"  \
gridOptionsOVS="--mem-per-cpu=64g --time=72:00:00" \
gridOptionsExecutive="--mem-per-cpu=24g --time=72:00:00" \
gridOptionsCORMHAP="--mem-per-cpu=10g --time=72:00:00" \
gridOptionsOBTMHAP="--mem-per-cpu=10g --time=72:00:00" \
gridOptionsUTGMHAP="--mem-per-cpu=10g --time=72:00:00" \
gridOptionsCOROVL="--mem-per-cpu=10g --time=72:00:00" \
gridOptionsOBTOVL="--mem-per-cpu=10g --time=72:00:00" \
gridOptionsUTGOVL="--mem-per-cpu=6g --time=72:00:00" \
gridOptionsRED="--mem-per-cpu=12g --time=72:00:00" \
gridOptionsOEA="--mem-per-cpu=12g --time=72:00:00" \
gridOptionsOVB="--mem-per-cpu=12g --time=71:00:00" \
gridOptionsCNS="--mem-per-cpu=12g --time=70:00:00" \
-nanopore-raw trimmed.q8_l2000.porechop.fastq.gz
```

Flye

```
flye --nano-raw trimmed.q8_l2000.porechop.fastq.gz --out-dir out_nano --genome-size 1.1g --threads 32 --
iterations 2
```

MaSuRCA - Config File

```
DATA
PE= pe 250 20
NANOPORE=/fullpath/nanopore.fa
END

PARAMETERS
EXTEND_JUMP_READS=0
GRAPH_KMER_SIZE = auto
USE_LINKING_MATES = 0
GRID_QUEUE=all.q
GRID_BATCH_SIZE=300000000
LHE_COVERAGE=30
LIMIT_JUMP_COVERAGE = 300
CA_PARAMETERS =  cgwErrorRate=0.15
KMER_COUNT_THRESHOLD = 1
CLOSE_GAPS=1
NUM_THREADS = 32
JF_SIZE = 200000000
SOAP_ASSEMBLY=0
END
```

wtdbg

```
p=19
S=4
reads=Reads.fasta.gz
```

```
wtdbg-1.2.8 -t 20 -i ${reads} -fo p_${p}_S_${S}  -p ${p} -S ${S} --tidy-reads 5000 --edge-min 2 --rescue-low-cov-
edges && wtdbg-cns -t 20 -i  p_${p}_S_${S}.ctg.lay -o p_${p}_S_${S}.ctg.lay.fas && assemblathon_stats_2.pl
p_${p}_S_${S}.ctg.lay.fas > p_${p}_S_${S}.ctg.lay.fas.assembly_stats
```
